# Great Ape Genomes Offer Insight into Human Evolution

**DOI:** 10.1371/journal.pbio.0020236

**Published:** 2004-07-13

Some primatologists have argued that to understand human nature we must understand the behavior of apes. In the social interactions and organization of modern primates, the theory goes, we can see the evolutionary roots of our own social relationships. In the genomic era, the age-old question, What makes us human? has become, Why are we not apes? As scientists become more adept at extracting biological meaning from an ever expanding repository of sequenced genomes, it is likely that our next of kin will again hold promising clues to our own identity.[Fig pbio-0020236-g001]

**Figure pbio-0020236-g001:**
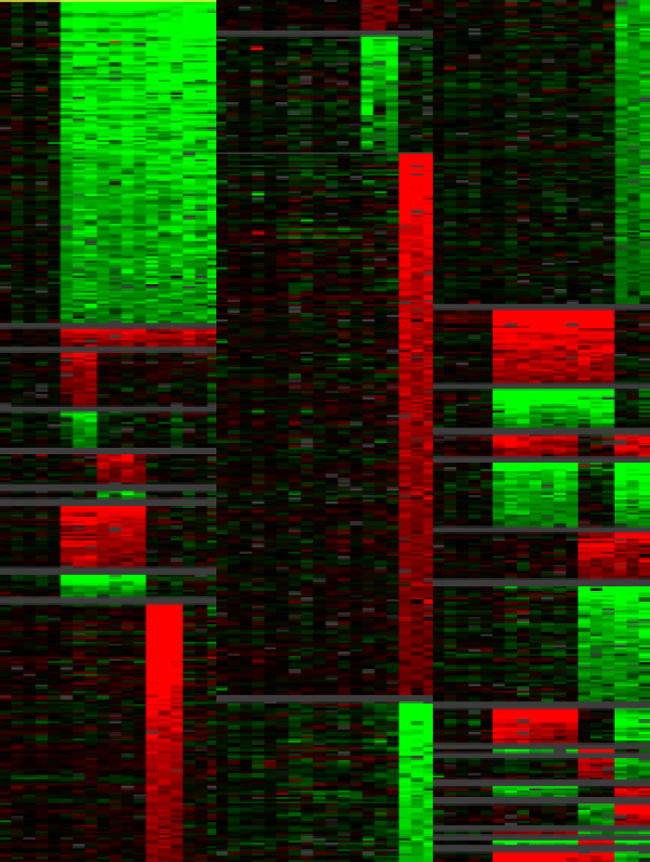
Lineage-specific gene gains and losses in humans and great apes

Many comparative genomics studies have looked to our more distant evolutionary relatives, such as the mouse and even yeast, to help interpret the human genome. Because the genomes of mice, yeast, and humans have diverged significantly since their last common ancestor—about 75 million years ago for mouse and human, and about 1 billion years ago for yeast and human—there are enough differences between the functional and nonfunctional regions to home in on biologically significant sequences, based on their similarity. Sequences that are similar, or conserved, in such divergent species are assumed to encode important biological functions. These comparative studies have successfully identified and characterized many human genes. And a similar approach comparing primate genomes can help scientists understand the genetic basis of the physical and biochemical traits that distinguish primate species. In this approach, however, rather than looking for genes that are shared across many species, scientists look for those that are unique to a species.

One of the primary agents of genome evolution is gene duplication. Duplicated genes provide the raw material for the generation of novel genes and biological functions, which in turn allow the evolution of organismal complexity and new species. (For more on duplicated genes, see the primer by Hurles in this issue.) James Sikela and colleagues set out to compare gene duplications between humans and four of our closest primate relatives to find the genetic roots of our evolutionary split from the other great apes. Collecting the DNA of humans, chimpanzees, bonobos, gorillas, and orangutans from blood and experimental cell lines, the researchers used microarray analysis to identify variations in the number of copies of individual genes among the different species. They analyzed nearly 30,000 human genes and compared their copy numbers in the genomes of humans and the four great apes.

Overall, Sikela and colleagues found more than 1,000 genes with lineage-specific changes in copy number, representing 3.4% of the genes tested. All the great ape species showed more increases than decreases in gene copy numbers, but relative to the evolutionary age of each lineage, humans showed the highest number of genes with increased copy numbers, at 134. Many of these duplicated human genes are implicated in brain structure and function.

The gene changes identified in the study, the authors conclude, likely represent most of the major lineage-specific gene expansions (or losses) that have taken place since orangutans split from the other great apes, some 15 million years ago. (Humans diverged from their closest cousins, the chimp and bonobo, roughly 5 million to 7 million years ago.) And because some of these gene changes were unique to each of the species examined, they will likely account for some of the physiological and morphological characteristics that are unique to each species. One cluster of genes that amplified only in humans was mapped to a genomic area that appears prone to instability in human, chimp, bonobo, and gorilla. This region, which corresponds to an ancestral region in the orangutan genome, has undergone modifications in each of the other descendent primate species, suggesting an evolutionary role. In humans, gene mutations in this region are also associated with the inherited disorder spinal muscular atrophy. This fact, along with the observation that there are human-specific gene duplications in this region, suggests a link between genome instability, disease processes, and evolutionary adaptation.

In their genome-wide hunt for gene duplications and losses in humans and great apes, Sikela and colleagues have highlighted genomic regions likely to have influenced primate evolution. With the impending release of the chimp genome and more primate sequences to follow, scientists can take advantage of both sequence-based and microarray-based genome information to wrest additional insights from our primate cousins and flesh out the details of the human story.

